# Cyclin-dependent kinase inhibitor p21 does not impact embryonic endochondral ossification in mice

**DOI:** 10.3892/mmr.2014.2889

**Published:** 2014-11-06

**Authors:** NOBUAKI CHINZEI, SHINYA HAYASHI, SHINGO HASHIMOTO, NORIYUKI KANZAKI, KENJIRO IWASA, SHUHEI SAKATA, SHINSUKE KIHARA, TAKAAKI FUJISHIRO, RYOSUKE KURODA, MASAHIRO KUROSAKA

**Affiliations:** Department of Orthopedic Surgery, Kobe University Graduate School of Medicine, Kobe, Hyogo 650-0017, Japan

**Keywords:** endochondral ossification, cell cycle, p21, p27, mouse embryo

## Abstract

Endochondral ossification at the growth plate is regulated by a number of factors and hormones. The cyclin-dependent kinase inhibitor p21 has been identified as a cell cycle regulator and its expression has been reported to be essential for endochondral ossification *in vitro*. However, to the best of our knowledge, the function of p21 in endochondral ossification has not been evaluated *in vivo*. Therefore, the aim of this study was to investigate the function of p21 in embryonic endochondral ossification *in vivo*. Wild-type (WT) and p21 knockout (KO) pregnant heterozygous mice were sacrificed on embryonic days E13.5, E15.5 and E18.5. Sagittal histological sections of the forearms of the embryos were collected and stained with Safranin O and 5-bromo-2′-deoxyuridine (BrdU). Additionally, the expression levels of cyclin D1, type II collagen, type X collagen, Sox9, and p16 were examined using immunohistochemistry, and the expression levels of p27 were examined using immunofluorescence. Safranin O staining revealed no structural change between the cartilage tissues of the WT and p21KO mice at any time point. Type II collagen was expressed ubiquitously, while type X collagen was only expressed in the hypertrophic zone of the cartilage tissues. No differences in the levels of Sox9 expression were observed between the two groups at any time point. The levels of cyclin D1 expression and BrdU uptake were higher in the E13.5 cartilage tissue compared with those observed in the embryonic cartilage tissue at subsequent time points. Expression of p16 and p27 was ubiquitous throughout the tissue sections. These results indicate that p21 may not be essential for embryonic endochondral ossification in articular cartilage of mice and that other signaling networks may compensate for p21 deletion.

## Introduction

The number of patients with cartilage disorders has been increasing globally (1). This is a complex issue compounded by the lack of a consensus on treatment; hence, the development of a conclusive therapy for cartilage disorders is necessary.

The process of cartilage tissue growth or longitudinal growth, also known as endochondral ossification, occurs within the long bones at the growth plate located between the epiphysis and the metaphysis (2,3). At the growth plate, chondrogenic differentiation occurs from the diaphyseal side toward the metaphyseal side and chondrocytes are arranged longitudinally in a columnar shape, forming a layered structure. These organized chondrocytes are divided into three principal zones: The resting zone, the proliferative zone and the hypertrophic zone. The resting zone consists of small and immature chondrocytes, which differentiate into more mature chondrocytes in the proliferating zone. Large chondrocytes are found in the hypertrophic zone, where they exhibit a 5–10-fold increase in size (3). In the proliferative zone, chondrocytes produce numerous significant extracellular matrix proteins (ECM), including type II collagen and aggrican, which are structurally essential to the growth plate (4). In the hypertrophic zone, chondrocytes produce type X collagen as they cease to proliferate (5). In the adjacent metaphyseal zone, chondrocytes undergo apoptotic cell death, attract blood vessels and lay down a true bone matrix within the cartilage matrix (2). Thus, endochondral ossification is characterized by the continual proliferation, differentiation, and growth arrest of chondrocytes, and is regulated by a number of factors and hormones (2).

Cyclin-dependent kinases (CDKs) are widely recognized as regulators of cell cycle progression and CDK activation is regulated by CDK inhibitors (CKIs) (6,7). p21 is a CKI which has been identified as a cell cycle regulator; its induction by p53 during the DNA damage-induced G_1_-phase checkpoint response inhibits CDK4 and CDK2 (8–10). Asada *et al* (11) reported that cytoplasmic p21 also acts as an inhibitor of apoptosis and clinical research focusing on p21 has been conducted in the fields of angiology and oncology (12,13). Furthermore, the association between the p53/p21 pathway and induced pluripotent stem cell generation has been established (14,15). In the field of regenerative medicine, Bedelbaeva *et al* (16) reported that a p21-knockout mouse strain was able to close ear hole wounds and displayed increased morphological and histological regenerative responses when compared with the wild-type (WT) mouse strain, providing a firm link between cell cycle checkpoint control and tissue regeneration. Several studies have reported that the expression of p21 is essential for chondrogenesis *in vitro* (17,18). Negishi *et al* (4) reported that the progression of chondrogenic differentiation requires the downregulation of CDK2-associated kinase activity with an increase in the levels of p21 protein, and the subsequent degradation of this protein via a proteasomal pathway. Despite studies which indicate the importance of p21, the original study of p21-knockout mice in 1995 described that these mice may develop normally (19). However, these results were reported strictly in adult mice from histological findings in areas such as muscles and vertebrae. Additionally, this study did not contain any information regarding the roles of p21 in the development of articular cartilage of limbs. The aim of the present study was to clarify the function of p21 in the embryonic endochondral ossification of articular cartilage in mice.

## Materials and methods

### Mouse breeding

All procedures were approved by the Animal Studies Committee at Kobe University, Kobe, Japan. p21 knockout mice (B6.129S6 (Cg)-Cdkn1atm1Led/J) were obtained from The Jackson Laboratory (Bar Harbor, ME, USA). All mice were housed in cages under pathogen-free conditions and were allowed unlimited access to water and food.

The mice were bred in the animal facility at Kobe University Graduate School of Medicine (Kobe, Japan). To generate heterozygous mice, homozygous p21 knockout (KO) and WT mice (C57BL/6J; CLEA Japan, Inc., Tokyo, Japan) were mated. Next, heterozygous mice were mated to obtain embryos from the two groups of mice: p21 KO and WT. A total of ten mice were used for each experiment.

### Tissue harvesting and decalcification

Pregnant heterozygous mice were anesthetized by an intraperitoneal injection of pentobarbital (50 mg/kg) and sacrificed by cervical disolcation at embryonic days E13.5, E15.5 and E18.5 (n=10 for each time point). Following collection of the embryos, the embryo forearms were dissected, fixed in 4% paraformaldehyde buffered with phosphate-buffered saline (PBS), decalcified with 10% formic acid and embedded in paraffin. Sagittal histological sections were cut at a thickness of 6 μm using a microtome and stained with Safranin O (Tokyo Chemical Industry Co., Ltd., Tokyo, Japan) and 5-bromo-2′-deoxyuridine (BrdU; BD Biosciences, San Jose, CA, USA). Tissue sections were also subjected to immunohistochemical and immunofluorescence analyses.

### BrdU labeling and staining

To confirm the cell cycle progression at the G_1_/S phase, pregnant mice were injected intraperitoneally with 200 μl BrdU and sacrificed by cervical dislocation 2 h later to obtain embryonic tissues. Staining was performed using a BrdU *In-Situ* Detection kit (BD Biosciences, Franklin Lakes, NJ, USA) according to the manufacturer’s instructions and the sections were examined using a BZ-8100 confocal microscope (Keyence, Osaka, Japan).

### Genotyping of mouse embryos

Genotypes were verified by polymerase chain reaction (PCR) analysis of tail-derived DNA. Genomic DNA was extracted using the DNeasy Blood & Tissue kit (Qiagen, Valencia, CA, USA). p21 deletion was confirmed by the presence of a 447-bp fragment unique to the mutant genotype, which was amplified with a p21-specific forward primer (5′-GTTGTCCTCGCCCTCATCTA-3′) and a mutant reverse primer (5′-CTGTCCATCTGCACGAGACTA-3′) (sequences provided by The Jackson Laboratory). WT alleles were confirmed by the presence of a 240 bp fragment amplified with the WT reverse primer (5′-GCCTATGTTGGGAAACCAGA-3′) and the p21-specific forward primer. DNA amplification was performed under the following PCR conditions: 94°C for 5 min, followed by 40 cycles of 94°C for 30 sec, 55°C for 30 sec and 72°C for 30 sec, and ending with 72°C for 2 min.

### Immunohistochemistry

De-paraffinized sections were digested with proteinase (Dako Retrieval Solution Ready-to-Use; Dako, Glostrup, Denmark) for 20 min and treated with 3% hydrogen peroxide (Wako Pure Chemical Industries, Osaka, Japan) to block endogenous peroxidase activity. In addition to BrdU staining, the expression of cyclin D1 was examined to determine cell cycle progression at the G_1_/S phase, as cyclin D1 is a G_1_/S phase-specific protein (20). Furthermore, the expression levels of p16, type II collagen, type X collagen, and Sox9 were examined. INK4 is a tumor suppressor protein which causes G_1_ phase cell cycle arrest and p16 is a known representative of the INK4 family (21). Type II collagen is the foundation for articular cartilage and hyaline cartilage and it has been established that type X collagen is produced by hypertrophic chondrocytes (4). Sox9 is essential for chondrocyte differentiation and cartilage formation (22). Tissue sections were treated overnight at 4°C in Can Get Signal immunostain solution A (Toyobo, Tokyo, Japan) and the following antibodies: rabbit anti-mouse cyclin D1 polyclonal antibody (1:50 dilution; Cell Signaling Technology, Inc., Danvers, MA, USA), rabbit anti-mouse p16 polyclonal antibody (1:50 dilution; Abbiotec LLC, San Diego, CA, USA), rabbit anti-mouse type II collagen polyclonal antibody (1:100 dilution; Cosmo Bio Co., Ltd, Tokyo, Japan), rabbit anti-mouse type II collagen polyclonal antibody (1:50 dilution; Cosmo Bio Co., Ltd) and rabbit anti-mouse Sox9 polyclonal antibody (1:100 dilution; Abcam, Cambridge, UK). Subsequently, the sections were treated with horseradish peroxidase (HRP)-conjugated goat anti-rabbit immunoglobulin anitibody (N-Histofine® Simple Stain Mouse MAX PO (R); Nichirei Bioscience, Tokyo, Japan) at room temperature for 30 min. The signal was developed as a brown reaction product using the peroxidase substrate 3,3′-diaminobenzidine (Histofine Simple Stain DAB solution; Nichirei Bioscience), and the sections were examined using a BZ-8000 Confocal microscope (Keyence, Osaka, Japan).

### Immunofluorescence

Deparaffinized sections were digested with proteinase (Dako Retrieval Solution Ready-to-Use) for 20 min and treated overnight at 4°C with the following antibodies in Can Get Signal immunostain solution A: Rabbit anti-mouse p27 polyclonal antibody (1:50 dilution; Santa Cruz Biotechnology, Inc.). The secondary antibodies used were goat anti-rabbit immunoglobulin Alexa Fluor 488 (1:200 dilution; Life Technologies, Carlsbad, CA, USA) for 30 min at room temperature. The nuclei were stained with DAPI and images were captured using a BZ-8000 confocal microscope (Keyence).

### Statistical analysis

Statistical analysis was performed using the SPSS version 16.0 software package (SPSS, Inc., Chicago, IL, USA). The differences in the percentages of cyclin D1 or BrdU-positive cells between the groups at each time point were analyzed using the Mann Whitney U-test. P<0.05 was considered to indicate a statistically significant difference. All data are expressed as the mean ±standard deviation.

## Results

### Cartilage tissue morphology in embryonic mice is not altered by p21 deficiency

**Figure 1:**
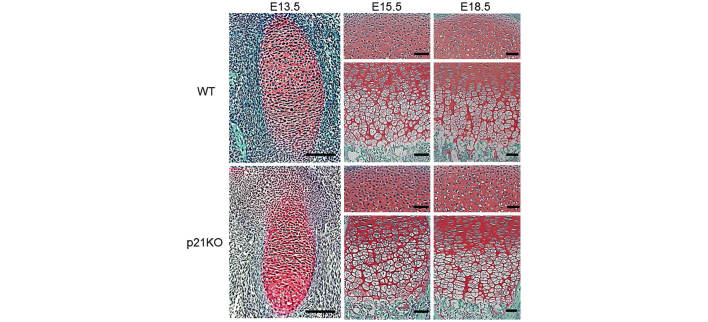
Safranin O staining in E13.5, E15.5 and E18.5 cartilage tissues. Upper panels, wild-type (WT) mice; lower panels, p21 knockout (KO) mice (scale bars, 100 μm).

To investigate the *in vivo* function of p21 in chondrogenesis, a histological analysis of cartilage tissues was performed at E13.5, E15.5 and E18.5. Safranin O staining revealed no structural changes at any time point between the embryonic cartilage tissues of WT and p21KO mice (Fig. 1). These results indicate that p21 deficiency does not alter the morphology of embryonic cartilage tissue in mice.

### Expression of ECM proteins and Sox9 is not altered by p21 deficiency

**Figure 2:**
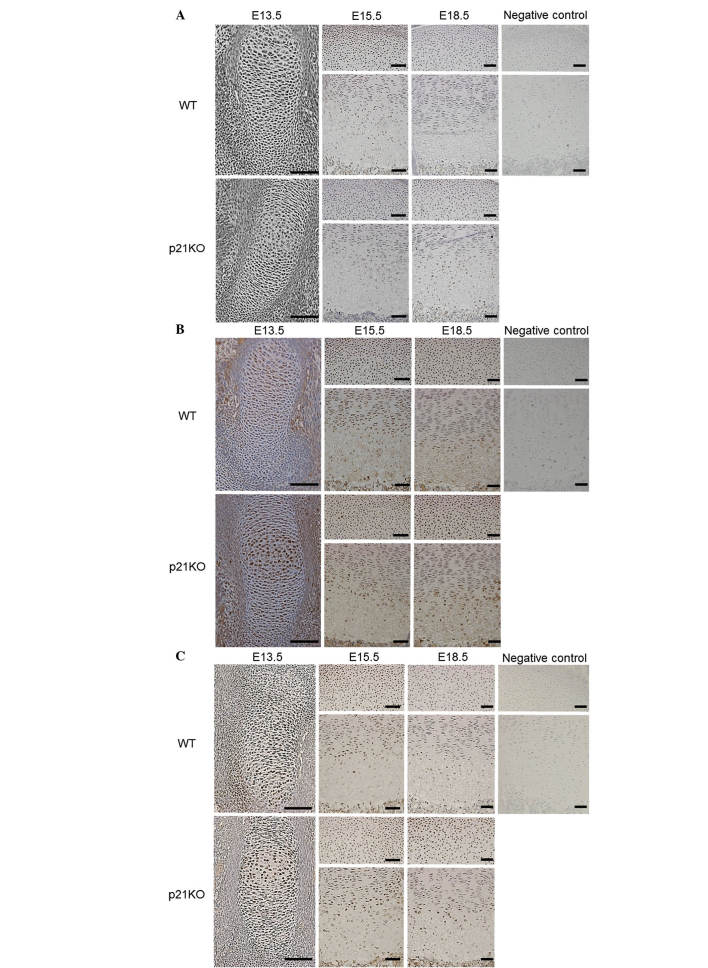
Expression of type II collagen, type X collagen and Sox9 in the mouse embryo. The expression of (A) type II collagen, (B) type X collagen and (C) Sox9 in E13.5, E15.5 and E18.5 cartilage tissues. Upper panels, wild-type (WT) mice; lower panels, p21 knockout (KO) mice (scale bars, 100 μm).

To investigate the *in vivo* function of p21 in ECM production, immunohistochemical analysis of cartilage tissue was performed at E13.5, E15.5, and E18.5. Type II collagen was expressed ubiquitously in the cartilage tissues. However, no differences were found between the embryonic cartilage tissues of WT and p21KO mice at any time point (Fig. 2A). Type X collagen was expressed in the hypertrophic zone. However, no differences were found between the embryonic cartilage tissues of WT and p21KO mice at each time point (Fig. 2B).

Immunohistochemical analysis revealed that the Sox9 expression levels of WT and p21KO mice did not differ at each time point (Fig. 2C). These results indicate that p21 deficiency does not affect ECM production in endochondral ossification.

### Chondrocyte proliferation is not altered by p21 deficiency

**Figure 3:**
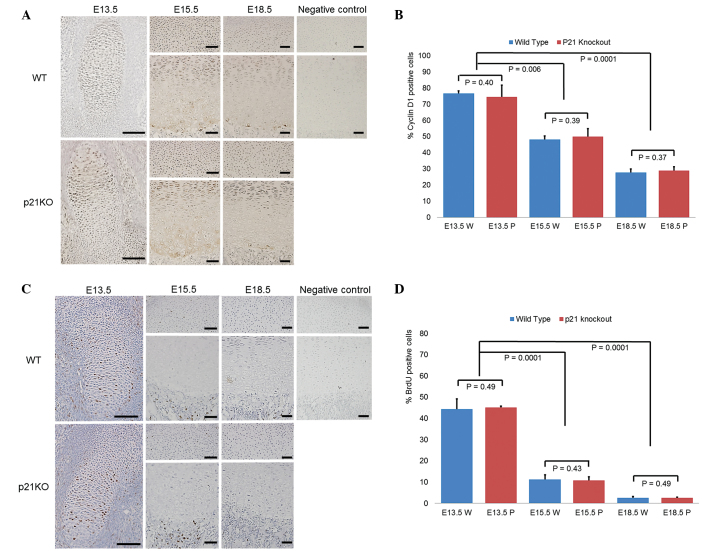
Cyclin D1 expression and 5-bromo-2′-deoxyuridine (BrdU) uptake in the mouse embryo. (A) The expression of cyclin D1 in E13.5, E15.5 and E18.5 cartilage tissues. Upper panels, wild-type (WT) mice; lower panels, p21 knockout (KO) mice (scale bars, 100 μm). (B) Quantitative analysis of cyclin D1 positive cells. Three micrographs of the growth plate were captured under ×40 magnification. The percentage of cyclin D1 positive cells was calculated as the ratio of the total number of cyclin D1 positive cells to the total number of chondrocytes. (C) The uptake of BrdU in E13.5, E15.5 and E18.5 cartilage tissues. Upper panels, WT; lower panels, p21KO (scale bars, 100 μm). (D) Quantitative analysis of BrdU positive cells. Three micrographs of the growth plate were captured under ×40 magnification. The percentage of BrdU positive cells was calculated as the ratio of the total number of BrdU positive cells to the total number of chondrocytes. P<0.05 was considered to indicate a statistically significant difference.

The main function of p21 is as a negative regulator of the G_1_/S transition, inducing ‘G_1_ arrest’ (23). Cyclin D1 staining was performed to evaluate the cell cycle progression at the G_1_ phase. Cyclin D1 forms complexes with CDK4 or CDK6, whose activity is required for G_1_/S phase transition (7). Although the expression levels of cyclin D1 were higher in the E13.5 cartilage tissue compared with those at subsequent time points, no differences were found between the WT and p21KO mice (Fig. 3A). Enumeration of cyclin D1-positive chondrocytes showed significant decreases at E15.5 and E18.5 compared with that of E13.5 (P<0.05). However, no significant inter-group differences were identified at any time point (P>0.05) (Fig. 3B).

BrdU staining was performed as BrdU is incorporated into proliferating cells (S phase) allowing it to be used to evaluate DNA replication (24). The uptake of BrdU appeared to be much higher in the E13.5 cartilage tissue compared with that at subsequent time points. However, no differences in BrdU uptake were observed between the WT and p21KO mice (Fig. 3C). The number of BrdU-positive chondrocytes was significantly reduced at E15.5 and E18.5 compared with the number at E13.5 (P<0.05). However, no significant differences between the two groups were observed at each time point (P>0.05) (Fig. 3D). These results indicate that p21 deficiency does not affect chondrocyte proliferation and that chondrocyte proliferation is naturally more active during the early embryonic period.

### Evaluation of the expression levels of p16

**Figure 4:**
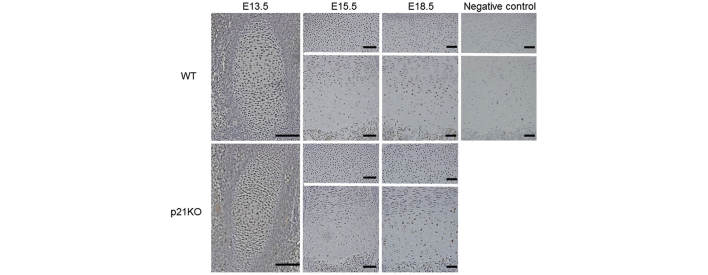
Expression of p16 in E13.5, E15.5 and E18.5 cartilage tissues. Upper panels, wild-type (WT) mice; lower panels, p21 knockout (KO) mice (scale bars, 100 μm).

INK4 is a tumor suppressor and cell cycle regulatory protein that acts during the G_1_ phase. p16 is a known representative of the INK4 family which interacts with CDK4 and CDK6, inhibiting their ability to interact with cyclin D (25). While p16 was expressed ubiquitously throughout the tissue sections, no differences were observed between the expression levels in the WT and p21KO mice (Fig. 4).

### Immunofluorescence evaluation of the expression of p27

**Figure 5:**
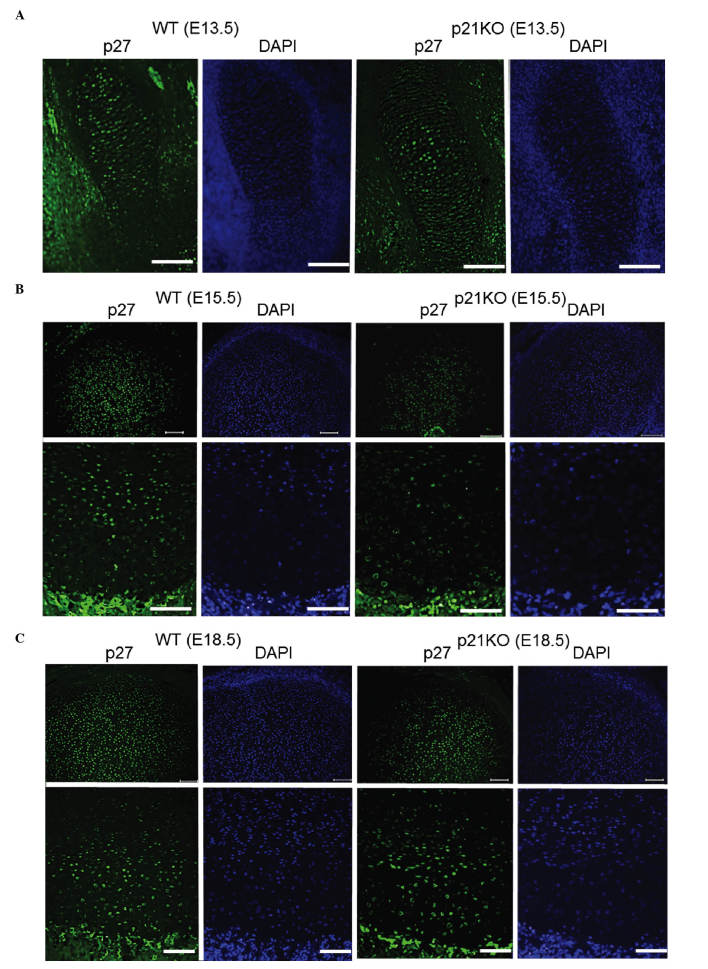
Immunofluorescence analysis of p27 expression in the mouse embryo with DAPI staining. The expression of p27 in (A) E13.5, (B) E15.5 and (C) E18.5 p21 knockout (KO) and wild-type (WT) mice (scale bars, 100 μm).

The expression of p27, one of the Cip/Kip family components, was evaluated by immunofluorescence and DAPI staining (Fig. 5A–C). p27 was ubiquitously expressed throughout the tissue sections, however, no differences were observed between the expression levels in the WT and p21KO mice (Fig. 5).

## Discussion

Accurate control of the cell cycle is essential for normal development, and CDKs are an integral part of cell cycle regulation (26). CDKs are specifically regulated by CKIs. Two distinct families of CKIs are known: Cip/Kip and INK4. The INK4 family consists of p15^INK4b^, p16^INK4a^, p18^INK4c^ and p19^INK4d^, which specifically inhibit the activity of G_1_-phase cyclin D-CDK4 and CDK6 (7,25). The Cip/Kip family, including p21^CIP1^, p27^KIP1^ and p57^KIP2^, controls a broader spectrum of cyclin-CDK complexes, including CDK2, CDK3, CDK4 and CDK6 (7,27). Although members of the CIP/KIP family possess a few similar functions, they also possess different functions which are determined by the differences in expression pattern and protein structure.

Previous studies have reported that p27 has an important role in endochondral ossification. In p27KO mice, multiple organ overgrowth has been observed (28) and Emons *et al* (29) reported that p27KO mice demonstrated a modest increase in body length. Furthermore, the expression levels of p27 mRNA were similar throughout the hypertrophic and resting/proliferative zones in adult mice. In the present study, p27 was ubiquitously expressed in the tissue sections assessed.

Previous studies have reported that p21 is expressed in the majority of organs and tissues during murine embryonic and postnatal development (30,31). In myogenesis, the muscle-specific transcription factor MyoD induces p21 expression in association with the terminal differentiation of muscles (31), suggesting that p21 has a crucial role in muscle development (6). In the current study, it was demonstrated that p21 deficiency did not alter the morphology of embryonic cartilage tissue in mice, although p21 has been shown to be expressed in the proliferative and hypertrophic zones in adult WT mice (32). Furthermore, p21 deficiency did not affect ECM production or Sox9 expression.

Additionally, the expression of cyclin D1 and INK4 family members was evaluated. The expression levels of cyclin D1 and p16 did not differ between the WT and p21KO mice. The similarity of the expression levels of cyclin D1 and p16 indicates that there are no differences in the remaining activities of G_1_-phase that the Cip/Kip family are involved in. The uptake of BrdU was observed to be much higher at E13.5 compared with E15.5 and E18.5. However, no differences in BrdU uptake were found between WT and p21KO mice, revealing that the rate of development was equivalent. Taking these results into consideration, p21 deficiency did not affect chondrocyte proliferation.

Therefore, the primary finding of the current study is that p21 may not be essential for embryonic articular chondrogenesis in mice. Negishi *et al* (26) reported that the reduction of endogenous p21 caused inhibition of early chondrogenic differentiation in ATDC5 cells, indicating that the p21 gene has an important role in this cellular process *in vitro* (26). However, the current study did not observe any marked changes *in vivo*, revealing a discrepancy in these findings. While the impact of these results within the general scientific community may not be great, the results obtained from the KO mice provide important information for the researchers in relevant fields. These results revealed that p21 deficiency does not impact the morphology, ECM formation, chondrocytic marker protein production, chondrocyte proliferation or cell cycle regulatory proteins in the developing cartilage.

It has been hypothesized that various complicated mechanisms control the expression and timing of the Cip/Kip family, which appear to possess important roles in development and growth regulation (6). Therefore, p21 deletion may be compensated by a complicated mechanism involving other networks. Further studies are required to gain insight into these phenomena. A clear understanding of these mechanisms may lead to the development of novel therapeutic strategies for cartilage disorders.

In conclusion, the current study revealed that p21 does not impact embryonic endochondral ossification in articular cartilage of mice. Furthermore, compensation for the lack of p21 function does not appear to be mediated by components of the Cip/Kip family, p27.
